# Psychological Well-Being in Italian Families: An Exploratory Approach to the Study of Mental Health Across the Adult Life Span in the Blue Zone

**DOI:** 10.5964/ejop.v13i3.1416

**Published:** 2017-08-31

**Authors:** Paul Kenneth Hitchcott, Maria Chiara Fastame, Jessica Ferrai, Maria Pietronilla Penna

**Affiliations:** a Department of Pedagogy, Psychology, Philosophy, University of Cagliari, Cagliari, Italy; Webster University Geneva, Geneva, Switzerland; University of Western Ontario, London, Canada

**Keywords:** adult development, aging families, psychological well-being, depression, blue zone

## Abstract

Self-reported measures of psychological well-being and depressive symptoms were examined across differently aged family members, while controlling for the impact of marital status and personal satisfaction about family and non-family relations. Twenty-one grandchildren (i.e., ages 21-36 years) were recruited with their parents (i.e., 48-66 years old) and grandparents (i.e., 75-101 years of age) in the ‘blue zone’ of Ogliastra, an Italian area known for the longevity of its inhabitants. Each participant was individually presented a battery of questionnaires assessing their lifestyle and several perceived mental health indices, including the Warwick-Edinburgh Mental Well-Being Scale (WEMWBS, Tennant et al., 2007), and the Center for Epidemiologic Studies Depression Scale (i.e., CES-D, Radloff, 1977). After assessing the level of concordance among adults sharing the same context, the Hierarchical Linear Modeling (HLM) approach was used to assess the nested dataset. It was found that family membership (i.e., grandchildren versus parents and grandparents) predicted the WEMWBS score but not the CES-D when the impact of marital status and personal satisfaction about social (i.e., family and non-family) ties was controlled for. Moreover, two separate repeated-measure Analyses of Variance (ANOVAs) documented similar level of personal satisfaction about social relationships across the three family groups. In conclusions, satisfying social ties with friends and family members together with an active socially oriented life style seems to contribute to the promotion of mental health in adult span.

Substantial research indicates that psychological and socio-cultural factors play a role in psychological well-being in late adulthood (e.g., Diener & Chan, 2011; Diener, Lucas, & Oishi, 2002). Subjective (or psychological) well-being refers to a multidimensional psychological construct that De Beni, Borella, Carretti, Marigo, and Nava (2007) defined in terms of one’s personal satisfaction (i.e., the level of self-appreciation with one’s past and present and the expectations of satisfaction in future), coping strategies (i.e., the ability to tackle problems in the daily life) and emotional competences (i.e., the ability to establish stable relationships with others and share emotional status with them).

Within a developmental psychology framework, there is evidence that aging is a complex phenomenon characterized by the continuous development of cognitive, metacognitive, social, and emotional abilities (Baltes, Lindenberger, & Staudinger, 1998), as a direct effect of many factors, including family and non-family relationships. In this regard, Antonucci and Akiyama (1987) proposed the ‘convoy model’ according to which individuals are immersed in dynamic social networks within which reciprocal exchanges shape an individual’s mental health across the entire life span. More recently, Margrett et al. (2011) found that greater perceived social support was associated with various outcomes including increased life satisfaction, greater extroversion and lower loneliness in a sample of American octogenarians and centenarians. Consistent with this, there is empirical evidence that positive family ties provide instrumental support for the maintenance of adequate levels of psychological well-being (e.g., Antonucci, Birditt, Sherman, & Trinh, 2011), especially in mid- to late-life (e.g., Ward, Spitze, & Deane, 2009). However, the impact of number of family ties on mental health of family members is quite controversial. Indeed, Peek and Lin (1999) found fewer depressive signs in people with a higher number of relatives in their social network than in individuals with a lower numbers of family members. In contrast, Haines, Beggs, and Hurlbert (2008) did not find any effect of the family proportion in shaping psychological well-being in adult life span. More recently, Fuller-Iglesias, Webster, and Antonucci (2015) conducted a longitudinal study on almost nine hundred young, middle-aged and elderly family members. The authors found that the size and composition of the family did not influence self-reported psychological well-being (i.e., occurrence of depressive signs) in young (i.e., 18-34 years) and middle-aged (i.e., 35-49 years) participants. In contrast, older adults (i.e., 50-80 years) receiving greater support from many family members reported fewer depressive signs over time, whereas young and middle-aged participants reporting a higher negative quality of family relationships (i.e., negative aspects such as conflict and burden) presented more depressive symptoms.

Growing evidence indicates that social support from friends is also relevant for the maintenance of physical and mental health in late adult span (e.g., Antonucci, 2001; Fuller-Iglesias, 2015; Fuller-Iglesias et al., 2015; Poon & Cohen-Mansfield, 2011). More specifically, recent studies have documented that active involvement in social activities provides elderly people with positive relationships that contribute to well-being (e.g., Everard, Lach, Fisher, & Baum, 2000; Litwin & Shiovitz-Ezra, 2006) and perceived physical health (Fuller-Iglesias, 2015). This seems crucial because further evidence highlights that depressive symptoms are highest among the oldest-old (Gostynski et al., 2002), who are most vulnerable to loneliness and a lack of social support (Dykstra, 2009). This is consistent with a wider literature (e.g., Cornwell & Laumann, 2015; Poon & Cohen-Mansfield, 2011) that confirms that social connectedness, even in late adulthood, can facilitate the access to social opportunities, improve self-esteem, increase physical activity and significantly reduce loneliness. One possible scenario is that the support of family members and friends across adult life span lowers psychological stress and depression, enhances cooperation and thereby reduces loneliness (Dykstra, 2009; Stroebe, Zech, Stroebe, & Abakoumkin, 2005).

Additionally, it has been documented that in collectivistic cultures (e.g., Japan), which reinforce interpersonal dependency, harmonious relationships with family and non-family networks supports psychological well-being (e.g., life satisfaction; Kitayama, Markus, & Kurokawa, 2000; Kwan, Bond, & Singelis, 1997).

The foregoing evidence is also consistent with findings from the Italian context, where the current study was conducted. For example, there are indications that more intense social support from family members and neighbors is associated with fewer depressive symptoms in later life (e.g., Carpiniello, Carta, & Rudas, 1989). In addition, de Belvis et al. (2008) found that fewer social contacts with friends is predictive of a decline in life quality among non-institutionalised Italian older people (i.e., individuals aged 60 years or more). Most recently, Fastame, Penna, and Hitchcott (2015) found that young adults and older people living in urban and rural area of Sardinia, a Mediterranean isle having a high prevalence of successful aging, were significantly less depressed than participants recruited in rural areas of Northern Italy, where the respondents were more socially isolated. Further details about this population are of interest. Compared to peers residing in Northern Italy, elderly people from the inner mountainous areas of Sardinia are more physically active, more involved in community social life, have lower loneliness and express greater confidence in their cognitive efficiency, as well as report higher personal satisfaction and emotional competence (Fastame & Penna, 2014; Fastame, Penna, Rossetti, & Agus, 2014). Moreover, Fastame, Penna, Rossetti, and Agus (2014) reported that higher self-perceived support from non-family members (i.e., respect and support from the younger generations) was associated to greater general psychological well-being, better self-efficacy and fewer depressive signs in late adulthood. According to Fastame, Penna, and Rossetti (2014), these positive outcomes may be because collectivist principles are highly valued in traditional Sardinian culture. Such speculation is consistent with previous studies (e.g., Martin, Hagberg, & Poon, 1997; Rook & Pietromonaco, 1987) indicating that the number and/or quality of social contacts represent a vital resource for older people.

The living arrangements of traditional Sardinian families typically promote collectivistic values; during late adulthood people are more likely to cohabit with adult children or to receive significant support from them (e.g., de Jong Gierveld & Van Tilburg, 1999; Hank, 2007). Although this suggests that familial networks may maintain the mental health of Sardinian adults, direct empirical support is lacking.

The current study was aimed at exploring the role of family membership (i.e., grandchild versus parent and grandparent) as a predictor of various mental health (i.e., psychological well-being and depressive symptoms) outcomes. Potentially confounding effects of marital status, and self-reported measures of personal satisfaction about family and non-family relationships were statistically controlled for.

An additional aim was to examine whether the mental health outcomes (i.e., psychological well-being, depressive measures), perceived physical health, satisfaction with family, and non-family ties varied across the three generations.

In the present study, psychological well-being was assessed using a recently validated Italian version (Gremigni & Stewart-Brown, 2011) of the Warwick-Edinburgh Mental Well-Being Scale (i.e., WEMWBS; Tennant et al., 2007). Depressive symptoms were determined using the Italian version of the Center for Epidemiologic Studies Depression Scale (i.e., CES-D, Radloff, 1977). Despite the wide use of these tools, to our knowledge, this is the first investigation using them to assess the impact of social networks on mental health. This would also appear to be the first study assessing mental health outcomes among family members recruited exclusively within the Sardinian ‘blue zone’, a longevity hot-spot (Poulain et al., 2004). In addition, to our knowledge, no previous studies reported the use of the WEMWBS (Tennant et al., 2007) to assess psychological well-being in very old Italian adults (i.e., over 82 years of age).

The lack of prior evidence limits speculation about certain aspects of this study; however, various hypotheses can be stated. First, it was hypothesized that family membership, together with marital status, perceived personal satisfaction about family and friend relationships would predict self-reported psychological well-being and depressive symptoms (e.g., Fastame, Penna, Rossetti, & Agus, 2014; Fastame, Penna & Hitchcott, 2015; Fuller-Iglesias et al., 2015; Kitayama et al., 2000). Second, mean depression scores were expected to be significantly lower than the Italian cut-off score as previously reported (e.g., Fastame, Penna, Rossetti, & Agus, 2014). Finally, in a collectivistic rural community where the current data were collected (e.g., Fastame, Penna, & Rossetti, 2014), similar level of personal satisfaction about family and non-family relationships were expected across the three family generations (i.e., grandchildren versus parents and grandparents) (e.g., Kwan et al., 1997; Kitayama et al., 2000).

## Method

### Participants

Sixty-three cognitively healthy community dwelling adults were recruited in twenty-one families residing in Arzana, a small village (i.e., approximate population 2500) located in the Sardinian ‘blue zone’ area of Ogliastra (Poulain et al., 2004), which is known for the longevity of its inhabitants and their successful aging (e.g., Fastame, Hitchcott, & Penna, 2015; Fastame, Penna, & Rossetti 2014). Participants were volunteers and were assigned to one of three family status groups, as appropriate: Grandchild, Parent, or Grandparent. Participants were required to meet all of the following inclusion criteria: (a) have been born and currently reside in Arzana; (b) live in a private house, with or without other members of the family; (c) be descendant of people that lived in that Sardinian area for at least two previous generations; (d) be cognitively healthy, that is, having a score ≥ 24/30 on the Mini-Mental State Examination test (MMSE; Folstein, Folstein, & McHugh, 1975); (e) each participant of the grandchildren group had to take part in the study with his/her parent and grandparent, that is, a granddaughter could participate only if her cognitively healthy mother and grandmother accepted to take part in the study (i.e., female lineal descendants), so did the young male adults with their fathers and grandfathers (i.e., male lineal descendants). Although these criteria restricted recruitment they were deemed necessary to isolate the influence of the Sardinian context and to be compatible with related previous research.

Volunteers were recruited through personal contacts of the third author and via direct appeals to local community groups. Respondents did not receive any reward (e.g., financial support) for their participation.

Table 1 illustrates the socio-demographic characteristics of the participants.

**Table 1:** Socio-Demographic Characteristics of the Sample

Characteristic	Young (children)		Middle-Aged (parents)		Very Old (grandparents)	
*n*	21		21		21	
Gender (*n*)						
Males	9		9		9	
Females	12		12		12	
Age (years)						
Range	21-36		48-66		75-101	
*M*	26.90		54.30		84.05	
*SD*	4.10		4.40		6.60	
Education (years)	Males	Females	Males	Females	Males	Females
0-8	0	1	4	6	9	12
> 8	9	11	5	6	0	0

Gender, χ^2^(2, *N* = 63) = 0.00, *p* = 1.00, was counterbalanced across the three family groups. Following De Beni et al. (2007), education was dichotomized according to low (i.e., 1-8 years) and high (> 8 years) levels. As one can see in Table 1, education was higher among Grandchildren relative to their Grandparents, χ^2^(2, *N* = 63) = 38.20, *p* < .001. This is not surprising. Younger generations from this region now have far greater opportunities to remain in formal education whereas older generations typically entered farming-related occupations following only basic formal education.

### Materials

Each participant first read and provided written informed consent to participate and were then presented the following questionnaires/test:

The Mini-Mental State Examination (MMSE; Folstein et al., 1975) is a pencil-and-paper screening test composed of 30 items assessing general cognitive efficiency (e.g., short-term and long-term memory, attention, visuo-motor coordination, spatial-temporal orientation). This tool provides a general cognitive efficiency score that according to the authors has to be corrected as a function of age and years of education. Internal consistency is expressed by a Cronbach's alpha ranging between .68 and .96. Test-retest reliability coefficients fall between .80 and .95. The level of sensitivity to correctly identify those individuals classified as cognitively impaired is .87, whereas the specificity coefficient which is used to identify those individuals who earlier were classified as cognitively intact is .82 (see Tombaugh & McIntyre, 1992).

The interview developed by Fastame and Penna (2012) was presented to collect information on lifestyle (e.g., time spent for outdoor leisure activities or gardening, type of hobbies, intake of medicine) and socio-demographic characteristics (e.g., age, years of education, marital status, people with which the respondents live).

Self-assessed personal satisfaction about family relationships index is a 10-point Likert-type scale containing two items assessing how much during the previous week the respondent was satisfied about his/her ties with family members. Zero indicated lack of satisfaction, whereas 10 denoted maximum satisfaction. The average score between the answers provided to the two items was calculated.

Self-assessed personal satisfaction about friendships index is a two-items measure asking participant to rate how much he or she was satisfied about his or her friendships and relations with the neighbors during the previous week along a 10-point Likert scale. The procedure used to calculate this index is identical to that adopted to rate the level of personal satisfaction about family relationships.

The CES-D (Radloff, 1977; Italian adaptation, Fava, 1983) contains 20 items measuring depressive signs experienced during the previous seven days on a four-point Likert scale (from 0, *never or rarely* to 3, *most days or every day*). The maximum total score is 60. A score ≥ 16 suggests the presence of clinical depression. In the current study the internal consistency was expressed by a Cronbach’s alpha of .83.

The WEMWBS (Tennant et al., 2007; Italian adaptation by Gremigni & Stewart-Brown, 2011) is composed of 12 items assessing perceived mental health during the two previous weeks, along a 5-point Likert scale ranging from 1 (i.e., *never*) to 5 (i.e., *always*). The maximum score is 60. The normative data for the Italian adult population (Gremigni & Stewart-Brown, 2011) documented that the mean score for the young adult 25-30 years old is 43.62 (*SD* = 5.74), the mean score for the middle-aged (i.e., 43-57 years old) group is 41.23 (*SD* = 4.5), whereas the mean score for the over 57 years old is 43.8 (*SD* = 6.7). In the current sample the internal consistency reliability is expressed by a Cronbach’s alpha of .73.

### Procedure

Each participant was interviewed individually in a quiet room of his/her home. To minimize fatigue among older people, all participants responded verbally. All instructions and statements contained in the questionnaires used in the current study were read aloud by the third author who also recorded responses. Typically testing sessions lasted ~60 minutes.

Within each family group, the oldest participant (i.e., grandparent) first completed the MMSE. If the score indicated no cognitive decline (≥ 24), the socio-demographic interview was presented. Following this the order of the remaining measures was counterbalanced according to a Latin square procedure. This was repeated for the parents and grandchildren. If signs of cognitive decline were found in any participant, the entire family triplet was excluded by the study. This resulted in the exclusion of only one triplet and was due to mild cognitive decline in the grandparent.

## Results

The interview by Fastame and Penna (2012) revealed that the marital status (i.e., being single vs. being engaged or married) was counterbalanced across the participants, χ^2^(1, *N* = 63) = 1.30, *p* = .26, but not across the three family groups, χ^2^(2, *N* = 63) = 22.2, *p* < .001. Indeed, as expected, 76.2% of the grandchildren were single, whereas 95.2% of their parents were married. Marital status was counterbalanced across the grandparents group, χ^2^(1, *N* = 21) = 0.05, *p* = .83, since 50% of the older participants were married.

The majority (90.4%) of participants reported sharing daily life with other family members (i.e., living with partner or parents or children vs. living alone). Specifically, 81% of the grandparents and 95% of the parents lived with their partner or children, and 95% of the young group lived with their partner or parents.

Intake of medicines (i.e., yes vs. no) was not counterbalanced across the three family groups, χ^2^(2, *N* = 63) = 22.23, *p* < .001. As expected, the use of drugs increased as a function of the age of the participants. Regular use of medicines was reported by 19% of young adults, 75% of their parents and 90.5% of their grandparents.

Attendance of socially-oriented activity (i.e., yes versus no) was counterbalanced across the participants, χ^2^(1, *N* = 63) = 2.70, *p* = .10. Overall, 62.3% of the respondents reported involvement in recreational/cultural or sport activities involving their social network. The breakdown across age groups was as follows - 66.7% of grandchildren, 76.2% of their parents and 38.1% of their grandparents. Sporting activities were the main pastime of grandchildren (i.e., 57.1%) and parents (i.e., 47.6%) relative to participation in recreational/cultural activities (i.e., 9.5% and 28.6%, respectively). As expected, grandparents reported higher participation in recreational/cultural activities (i.e., 23.8%) relative to sporting ones (i.e., 14.3%). A high proportion (73%) of participants reported involvement in gardening/farming activities, χ^2^(1, *N* = 63) = 13.35, *p* < .001. This was expected considering agro-pastoral nature of this region of Sardinia.

As participants were enrolled within the same families, the degree of intra-family correlations between the various mental health indexes (i.e., psychological well-being, personal satisfaction about family and non-family ties, depression) were calculated in terms of Intraclass Correlation Coefficients (ICC), using the methods suggested by Donner and Koval (1980). Asymptotic variance for the estimate of ICCs was also obtained. Following Cohen (1988), concordance was considered high if ICC values exceeded 0.60, moderate if in the range 0.30-0.60 and low below 0.30. Coefficients for the WEMWBS, ρ = .12, *F*(2,42) = 1.15, *p* = .33, and CES-D measures, ρ = .18, *F*(2,42) = .26, *p* = .77, family resemblance were low. Similar coefficients were found when ICC was calculated with regard to personal satisfaction about family relationships, ρ = .05, *F*(2,42) = .75, *p* = .48, and personal satisfaction about non-family ties, ρ = .09, *F*(2,42) = 1.5, *p* = .24.

Next, a multilevel modeling approach was used to investigate whether family membership (i.e., grandchildren, parents or grandparents enrolled within the same family) predicted self-reported psychological well-being (i.e., WEMWBS and CES-D, respectively), while controlling for the effect of marital status (i.e., single vs. engaged/married), personal satisfaction about family relationships and personal satisfaction about non-family relationships. A random intercept was included, assuming simply that intercepts for the relationships between family members and psychological well-being (when controlling for marital status, and personal satisfaction about family relationships and personal satisfaction about non-family relationships) vary over families. The linear model also included random slopes, because it was hypothesized that psychological well-being scores could vary across grandchildren, parent and grandparent groups. Maximum likelihood was the method of estimation used to produce the parameter estimates.

The relationship between family membership and WEMWBS scores did not show any significant variance in intercepts across participants when the effect of the covariates was controlled for, var(u0j) = 2.07, χ^2^(1, *N* = 63) = 0.00, *p* > .05. Therefore, one can conclude then that the intercepts for the relationship between family membership and perceived psychological well-being (when controlling for the impact of personal satisfaction about family and non-family relationships and marital status) did not vary significantly across the different families. In addition, the slopes did not vary significantly across participants, var(u1j) = 2.68, χ^2^(1, *N* = 63) = 0.00, *p* > .05. Moreover, adding the covariance between slopes and intercepts did not make a significant difference to the model, cov(u0j, u1j) = 1.3, χ^2^(8, *N* = 63) = 2.26, *p* > .05. By allowing the intercepts to vary over families, WEMWBS scores were found to be significantly predicted by family membership, *F*(2, 26.8) = 3.56, *p* = .04, personal satisfaction about family relationships, *F*(1, 41.2) = 29.2, *p* < .001, personal satisfaction about non-family relationships, *F*(1, 29.5) = 11.3, *p* = .002, and marital status (i.e., single vs. engaged/married), *F*(1, 39.6) = 6.3, *p* = .02. Controlling for the effect of the covariates, the person-level intercept for grandchildren had an estimated mean in the WEMWBS scale of 21.67 (95% CI for the difference was .53 to 4.67), that was significantly higher than that for their grandparents, 19.08, 95% CI [9.9, 28.2], *t* = 2.61, *p* =.016. In contrast, the intercept for the parents was 20.48, that was similar to the intercept for the older family members, *t* = 1.3, *p* = .22, 95% CI for the difference was -.86 to 3.67. Ignoring the effect of family membership, every one point increase in the personal satisfaction about family relationship score was associated with a 1.62 to 3.6 increase in WEMWBS score while the effects of the personal satisfaction about non-family relationships and civil status were controlled for, *t* = 5.4, *p* < .001. Similarly, the increase in the mean score of the person-level slope for personal satisfaction about non-family relationship was associate with a .30 to 1.24 increase in the WEMWBS score, *t* = 3.36, *p* = .002, controlling for the impact of civil status. Finally, the mean WEMWBS score decreased 0.51 to 4.70 points from engaged/married participants to single ones, *t* = -2.50, *p* = .02, while controlling for personal satisfaction about family and non-family relationships. In conclusion, family membership significantly predicted WEMWBS index after controlling for the effect of personal satisfaction about family and non-family relationships and civil status, *b* = - 1.21, *t*(63) = - 2.24, *p* = .03. The same analyses were replicated using CES-D score as dependent variable. The relationship between family membership and depression did not show significant variance in intercepts across participants, var(u0j) = 9.15, χ^2^(1, *N* = 63) = 1.82, *p* > .05, suggesting that similar CES-D scores were found across the families. In addition, the slopes did not vary across participants, var(u1j) = 5.12, χ^2^(1, *N* = 63) = 0.00, *p* > .05, and the slopes and intercepts did not significantly covary, cov(u0j, u1j) = 4.4, χ^2^(8, *N* = 63) = 6.60, *p* > .05. Personal satisfaction about family relationships, *F*(1, 45.2) = 15.4, *p* < .001, and personal satisfaction about non-family relationships, *F*(1, 37.4) = 6.51, *p* = .01, both did significantly predict self-reported depression, whereas family membership, *F*(2, 28.5) = .75, *p* = .48, and civil status, *F*(1, 39.3) = .003, *p* = .96, did not significantly predict CES-D score. Ignoring the impact of family membership, every one point increase in the personal satisfaction about family relationship score was associated with a 1.28 to 3.98 decrease in CES-D score while the effects of the personal satisfaction about non-family relationships and civil status were controlled for, *b* = - 2.63, *t*(45.2) = - 3.9, *p* < .001. Furthermore, the increase in the mean score of the personal satisfaction about non-family relationships score was associate with a .17 to 1.48 decrease in the CES-D score, *b* = -.82, *t*(37.4) = - 2.5, *p* = .01.

Finally, two repeated-measures Analyses of Variance (ANOVAs) were conducted to explore the effect of family membership (i.e., grandchildren vs. parents and grandparents) on personal satisfaction about family and non-family relationships. Grandchildren reported scores similar to those of their parents and grandparents on personal satisfaction about family relationship, *F*(2,40) = .75, *p* = .48, and personal satisfaction about non-family, *F*(2,40) = 1.5, *p* = .24, scales. Table 2 illustrated the mean scores reported by the three family groups on the afore mentioned measures as well as on the WEMWBS and CES-D scales (while controlling for the effect of personal satisfaction about family and non-family relationships measures).

**Table 2:** Mean (i.e., M) Scores Relative to the Warwick-Edinburgh Mental Well-Being Scale (i.e., WEMWBS), Depressive Signs (i.e., CES-D), After Controlling for Perceived Satisfaction About Family (i.e., Satisf. Family), and Perceived Satisfaction About Nonfamily (i.e., Satisf. Friendships) Measures

Measure	Grandchildren		Parents		Grandparents	
	*M*	*SD*	*M*	*SD*	*M*	*SD*
WEMWBS	47.8	4.7	47.8	4.7	46.1	4.1
CES-D	11.0	6.8	11.5	5.5	12.2	6.0
Satisf. Family	8.6	0.9	8.5	0.9	8.8	0.7
Satisf. Friendships	7.6	1.5	6.8	2.3	6.9	1.3

## Discussion

The main aim of the current study was to explore whether family membership (i.e., grandchildren versus parents and grandparents) is a predictor of various measures of mental health (i.e., psychological well-being and depressive signs) in Sardinian families residing in the ‘blue zone’ of Arzana (Poulain et al., 2004), while controlling for the effect of marital status and perceived personal satisfaction about family and non-family relationships.

Overall, several meaningful findings emerged from this preliminary investigation. First, considering that the respondents were raised in the same context (i.e., families living at Arzana), the calculation of ICC provided meaningful information, because it represents the proportion of the total variability in the outcome that is attributable to the families. With respect to the assessment of several mental health self-reported measures (i.e., depression, psychological well-being, personal satisfaction about family and non-family relationships) ICC values (i.e., < .30) reflected a marginal degree of similarity among participants enrolled within the same families (see Cohen,1988). Then, taking into account that family context had a little effect on the respondents, an HLM method was warranted to deal with the nested design of the dataset, since each participant belonged to one unique family. Therefore, the people tested for the study was the Level 1 variable, whereas the family to which they belonged represents the Level 2 variable. Overall, considering the main outcomes coming from the proposed models, one can conclude that, as suggested by the wider literature (e.g., Fuller-Iglesias et al., 2015; Poon & Cohen-Mansfield, 2011), the current findings document that apart from marital status, personal satisfaction about exchanges with family and non-family members, family membership played a crucial role for the assessment of WEMWBS score. Indeed, young adults reported greater life satisfaction than their grandparents. In contrast, CES-D score was significantly predicted only by the level of personal satisfaction about family and non-family relationships. Moreover, as hypothesized, similar level of satisfaction about family and non-family relationships were found across the three family generations (e.g., Kwan et al., 1997; Kitayama et al., 2000).

Various findings are also consistent with the lifestyle information provided by the respondents. Most participants attended socially oriented leisure activities (i.e., recreational/cultural and sport practices), that represent a good opportunity to establish or strength their friendships, reduce loneliness and improve physical activity. Such outcomes are known to be beneficial to mental health (Cornwell & Laumann, 2015). Furthermore, in agreement with previous studies conducted in the Sardinian ‘blue zone’ (e.g., Fastame, Penna, Rossetti, & Agus, 2014), an active lifestyle was characteristic of the sample and few participants were sedentary. In addition to spending time in socially oriented activities, the participants were often involved in gardening or farming. As suggested by previous authors, gardening has a positive impact on psychological well-being in adulthood (Heliker et al., 2001) and high participation is characteristic of rural areas of Ogliastra (Fastame, Penna, & Rossetti, 2014).

Moreover, in line with previous findings (e.g., de Jong Gierveld & Van Tilburg, 1999; Fastame, Hitchcott, & Penna, 2015; Hank, 2007; Kitayama et al., 2000; Kwan et al., 1997) the current outcomes also show that family support promoted psychological well-being across adult life span (Dykstra, 2009; Margrett et al., 2011). Most participants lived with other family members and reported a very high level of personal satisfaction (i.e., mean score ≥ 8.5) about their relationships with relatives. As suggested by Fuller-Iglesias et al. (2015), collectivistic contexts, such as that of Ogliastra (e.g., Fastame, Penna, & Rossetti, 2014), engender both high personal satisfaction about one’s relationships with his or her relatives and friends. This may represent a significant resource for the protection of mental health. In the present study, WEMWBS and CES-D scores suggested high levels of mental health were present across the three generations of the families studied. Levels of depressive symptoms were low relative to normative data and diagnostic cut-offs for the Italian population. Similarly, levels of psychological well-being (i.e., WEMWBS) exceeded normative levels for the Italian population (Gremigni & Stewart-Brown, 2011). Therefore, unlike the findings reported by Fuller-Iglesias et al. (2015), the current outcomes suggest that the socio-cultural context of the Sardinian ‘blue zone’ is a protective factor for mental health throughout adult life span.

To summarize, the present observations build on previous reports that superior mental health among inhabitants of the Sardinian ‘blue zone’ can be traced to the impact of various psychosocial factors (e.g., Fastame & Penna, 2012; Fastame, Penna, & Hitchcott, 2015). The present study identifies a novel effect, arising from close, satisfying interpersonal relationships and suggests this has a pervasive influence across adult life span (e.g., Carpiniello et al., 1989; Fastame, Penna, Rossetti, & Agus, 2014). However, this is a very preliminary investigation, therefore it presents some limitations. First, the sample size is small and the participants were recruited only in the Sardinian ‘blue zone’. Although the tendency to look after and support elderly people is typical of Mediterranean countries (e.g., de Jong Gierveld & Van Tilburg, 1999; Hank, 2007) further investigation with larger samples of differently aged adults from other regions is necessary. This would help determine whether highly context-specific combinations of psychosocial factors, including the characteristics connected with family and non-family networks, promote successful aging within the Sardinian ‘blue zone’. However, if the current findings can be generalized, it would suggest that this population may represent a valuable resource for the investigation of successful ageing. Second, to our knowledge it is the first involving use of the WEMWBS scale among Italian the oldest-old (i.e., over 80 years old) participants. An obvious limitation is that the stringent inclusion criteria constrained the number of families that could be recruited from such a small village (i.e., population < 2500 inhabitants). While the strict inclusion criteria enhance the reliability of the findings cautious interpretation of the findings is essential. Third, the impact of social desirability on the self-assessment of psychological well-being and depressive signs was not investigated. Future research has to address this issue, because a recent trend of research (Fastame & Penna, 2012; Fastame, Hitchcott, & Penna, 2017; Hitchcott, Fastame, Penna, & Agus, 2016) suggests that socially desirable responding style should be taken into consideration when self-rated mental health measures of mental health are administered, because social desirability scores tend to increase with age and in women.

In conclusion, replication of the current findings, would promote understanding of mental health and successful ageing and, possibly, shape health service policies aimed at promoting quality of life in Italian adults.
